# Outcomes of left atrial appendage occlusion vs. non-vitamin K antagonist oral anticoagulants in atrial fibrillation

**DOI:** 10.1007/s00392-021-01983-z

**Published:** 2022-01-07

**Authors:** Wern Yew Ding, José Miguel Rivera-Caravaca, Elnara Fazio-Eynullayeva, Paula Underhill, Dhiraj Gupta, Francisco Marín, Gregory Y. H. Lip

**Affiliations:** 1 Liverpool Centre for Cardiovascular Science, University of Liverpool and Liverpool Heart and Chest Hospital, Liverpool, UK; 2 Department of Cardiology, Hospital Clínico Universitario Virgen de La Arrixaca, University of Murcia, Instituto Murciano de Investigación Biosanitaria (IMIB-Arrixaca), CIBERCV, Murcia, Spain; 3 TriNetX LLC., Cambridge, MA USA; 4 TriNetX LLC., London, UK; 5 Aalborg Thrombosis Research Unit, Department of Clinical Medicine, Aalborg University, Aalborg, Denmark

**Keywords:** Atrial fibrillation, Left atrial appendage occlusion, Non-vitamin K antagonist oral anticoagulant, Outcome, All-cause mortality, Thromboembolism

## Abstract

**Background:**

The effects of left atrial appendage (LAA) occlusion compared to non-vitamin K antagonist oral anticoagulant (NOAC) therapy in patients with atrial fibrillation (AF) remain unknown.

**Aims:**

We aimed to evaluate the outcomes in patients with AF who received LAA occlusion vs. NOAC therapy.

**Methods:**

We utilised data from TriNetX which is a global federated health research network currently containing data for 88.5 million patients. ICD-10 codes were employed to identify AF patients treated with either LAA occlusion or NOAC between 1st December 2010 and 17th January 2019. Clinical outcomes of interest were analysed up to 2 years.

**Results:**

108,697 patients were included. Patients who underwent LAA occlusion were younger, more likely to be white Caucasian and male, had a greater incidence of comorbidities, and were less likely to be prescribed other cardiovascular medications. Using propensity score matching, the risk of all-cause mortality was significantly lower among patients who received LAA occlusion compared to NOAC therapy [1.51% vs*.* 5.60%, RR 0.27 (95% CI 0.14–0.54)], but there were no statistical differences in the composite thrombotic or thromboembolic events [8.17% vs*.* 7.72%, RR 1.06 (95% CI 0.73–1.53)], ischaemic stroke or TIA [4.69% vs*.* 5.45%, RR 0.86 (95% CI 0.54–1.38)], venous thromboembolism [1.66% vs*.* 1.51%, RR 1.10 (95% CI 0.47–2.57)] and intracranial haemorrhage [1.51% vs*.* 1.51%, RR 1.00 (95% CI 0.42–2.39)].

**Conclusion:**

Overall, LAA occlusion might be a suitable alternative to NOAC therapy for stroke prevention in patients with AF.

**Graphical abstract:**

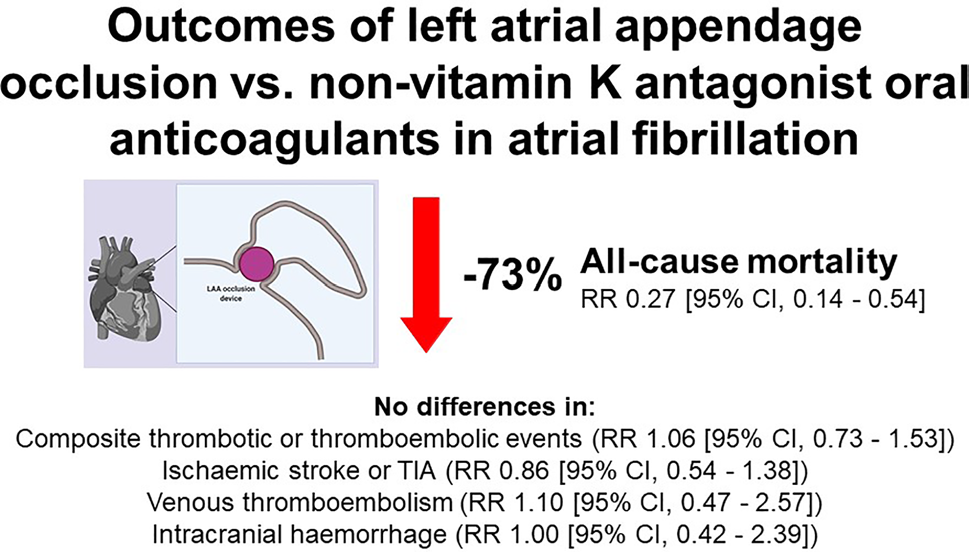

**Supplementary Information:**

The online version contains supplementary material available at 10.1007/s00392-021-01983-z.

## Introduction

Vitamin K antagonists (VKAs), such as warfarin, have traditionally been used to prevent thromboembolic complications in patients with atrial fibrillation (AF). However, warfarin is limited by a narrow therapeutic window, an increased risk of bleeding, and possible interactions with other drugs and food. Over the past decade, non-vitamin K antagonist oral anticoagulants (NOACs) have been shown in several landmark randomised controlled trials (RCTs) to be superior to warfarin, with comparable efficacy for stroke prevention but reduced risk of serious bleeding [1–5]. Nonetheless, there remains a proportion of patients who are unsuitable for anticoagulation due to conditions that predispose to a very high-risk of life-threatening bleeding, severe side effects or pregnancy (1st and 3rd trimester).

More recently, left atrial appendage (LAA) occlusion has emerged as a potential alternative therapy for stroke prevention in AF [6]. Findings from the National Cardiovascular Data Registry LAA occlusion registry of 38,158 procedures performed between January 2016 and December 2018 showed that the procedure was associated with a success rate of 98.1% to achieve a less than 5 mm leak, while maintaining a low incidence of major in-hospital adverse events (2.2%) [7]. Furthermore, LAA occlusion may result in better long-term outcomes compared to warfarin [8]. However, there are limited studies comparing the effects of LAA occlusion to NOAC therapy. To date, the PRAGUE-17 trial remains the only prospective RCT that has addressed this topic [9]. Herein, we aimed to evaluate the outcomes in patients with AF who received LAA occlusion vs. NOAC therapy.

## Methods

In this study, we used data from TriNetX, a global federated health research network with real-time updates of anonymised electronic medical records, predominantly in the United States. The network currently comprised 66 health-care organisations, including academic medical centres, speciality physician practices and community hospitals, and contains data for around 88.5 million patients across 11 countries. Further details about TriNetX processes and standardisation of data are in the Supplementary Materials.

We performed a search using ICD-10 codes (details in Supplementary Materials) on the 26th of January 2021 for patients with AF who were treated with either LAA occlusion or NOAC between 1st of December 2010 and 17th of January 2019. All patients who were aged over 18 years and received either surgical/catheter LAA occlusion or NOAC therapy alone were included. Exclusion criteria were rheumatic heart disease and acute rheumatic fever.

Data on baseline demographics, comorbidities (e.g. hypertension, coronary artery disease, diabetes mellitus, heart failure, previous stroke, peripheral vascular disease, prior gastrointestinal haemorrhage and prior intracerebral haemorrhage) and medication use (e.g. anticoagulants, antiplatelets, beta-blockers, calcium channel blockers, anti-arrhythmic drugs, angiotensin-converting enzyme inhibitors, angiotensin receptor blockers and diuretics) were collected.

Clinical outcomes of interest were all-cause mortality, composite thrombotic and thromboembolic events, ischaemic stroke or transient ischaemic attack (TIA), venous thromboembolism and intracranial haemorrhage. These were recorded from 30 days after treatment until a pre-specified follow-up duration of 2 years and defined using ICD-10 codes.

As a federated network, research using TriNetX does not require ethical approval. To comply with legal frameworks and ethical guidelines guarding against data re-identification, the identity of participating health-care organisations and their individual contribution to each dataset are not disclosed. The TriNetX platform only uses aggregated counts and statistical summaries of de-identified information. No protected health information or personal data is made available.

### Statistical analysis

Continuous variables were expressed as mean and standard deviation (SD), and tested for differences with independent-sample *t* test. Categorical variables were expressed as absolute frequencies and percentages, and tested for differences with Chi-squared test. Propensity score matching (PSM) in a 1:1 ratio was performed using logistic regression with nearest-neighbour matching at a tolerance level of 0.01 and difference between propensity scores of equal or less than 0.1 (i.e. caliper) for age, sex, race, hypertension, hypercholesterolaemia, coronary artery disease, diabetes mellitus, heart failure, chronic obstructive pulmonary disease, previous stroke, peripheral vascular disease, prior gastrointestinal haemorrhage, prior intracerebral haemorrhage, anticoagulant use, antiplatelet therapy, beta-blocker, calcium channel blocker use, anti-arrhythmic drug therapy, angiotensin-converting enzyme inhibitor use, angiotensin receptor blocker use and diuretic therapy. Covariate balance between groups was assessed using standardised mean differences, with a value below 0.1 indicating minimal differences between groups. Plots of Kaplan–Meier curves for study outcomes were created and survival distributions were assessed using log-rank test. Relative risk (RR) with 95% confidence intervals (CIs) was calculated. To validate our findings, an additional analysis was performed to compare the effects of LAA occlusion vs. VKA therapy in a separate subgroup of PSM patients using the method described above. No imputations were made for missing data. All *p *values were two-sided, and the significance level was set at 0.05. Statistical analysis was performed using the TriNetX Analytics function in the online research platform.

## Results

We included 108,697 patients with AF who were treated with LAA occlusion (*n* = 699) or NOAC therapy (*n* = 107,998). Compared to patients on NOAC therapy, those who received treatment with LAA occlusion were younger, more likely to be white Caucasian and male, and had a greater incidence of comorbidities including hypertension, hypercholesterolaemia, coronary artery disease, diabetes mellitus, heart failure, chronic obstructive pulmonary disease, previous stroke, peripheral vascular disease, prior gastrointestinal haemorrhage and prior intracerebral haemorrhage (Table 1). Patients in the LAA occlusion group were less likely to be prescribed other cardiovascular medications such as beta-blockers, calcium channel blockers, anti-arrhythmic drugs, angiotensin-converting enzyme inhibitors, angiotensin receptor blockers and diuretics. The most commonly used NOAC was apixaban, followed by rivaroxaban, dabigatran and edoxaban. After PSM, there were a total of 1,322 patients in both groups with comparable baseline characteristics (Table 2).

**Table 1:** Baseline characteristics of patients with LAA occlusion vs. NOAC therapy before propensity score matching

	LAA occlusion (*n* = 699)	NOAC (*n* = 107,998)	*p* value	SMD
Age (years), mean (± SD)	70.0 ± 10.6	71.0 ± 12.0	0.031	0.087
Female sex, *n* (%)	240 (34.3%)	49,718 (46.0%)	< 0.001	0.240
White Caucasian, *n* (%)	625 (89.4%)	90,814 (84.1%)	< 0.001	0.158
Comorbidities, *n* (%)				
Hypertension	496 (71.0%)	63,165 (58.5%)	< 0.001	0.263
Hypercholesterolaemia	382 (54.6%)	33,123 (30.7%)	< 0.001	0.500
Coronary artery disease	407 (58.2%)	19,704 (18.2%)	< 0.001	0.903
Diabetes mellitus	246 (35.2%)	23,695 (21.9%)	< 0.001	0.297
Heart failure	257 (36.8%)	20,576 (19.1%)	< 0.001	0.403
Chronic obstructive pulmonary disease	111 (15.9%)	12,209 (11.3%)	< 0.001	0.134
Previous stroke	53 (7.6%)	4,629 (4.3%)	< 0.001	0.140
Peripheral vascular disease	55 (7.9%)	3,583 (3.3%)	< 0.001	0.199
Prior gastrointestinal haemorrhage	48 (6.9%)	1,609 (1.5%)	< 0.001	0.271
Prior intracerebral haemorrhage	15 (2.1%)	452 (0.4%)	< 0.001	0.154
Medications, *n* (%)				
Anticoagulants				
Apixaban	NA	55,490 (51.4%)	NA	NA
Dabigatran		12,538 (11.6%)		
Edoxaban		427 (0.4%)		
Rivaroxaban		40,111 (37.1%)		
Antiplatelets				
Aspirin	190 (27.2%)	3,546 (3.3%)	< 0.001	0.705
Clopidogrel	56 (8.0%)	641 (0.6%)	< 0.001	0.372
Beta-blockers	219 (31.3%)	66,559 (61.6%)	< 0.001	0.638
Calcium channel blockers	117 (16.7%)	36,900 (34.2%)	< 0.001	0.408
Anti-arrhythmic drugs	164 (23.5%)	40,509 (37.5%)	< 0.001	0.309
ACE-inhibitors	98 (14.0%)	25,372 (23.5%)	< 0.001	0.244
ARB	59 (8.4%)	18,080 (16.7%)	< 0.001	0.252
Diuretics	159 (22.7%)	42,439 (39.3%)	< 0.001	0.364

*ACE* angiotensin-converting enzyme, *ARB* angiotensin receptor blocker, *SD* standard deviation, *SMD* standardised mean difference

**Table 2:** Baseline characteristics of patients with LAA occlusion vs*.* NOAC therapy after propensity score matching

	LAA occlusion (*n* = 661)	NOAC (*n* = 661)	SMD
Age (years), mean (± SD)	69.9 ± 10.8	69.2 ± 12.6	0.063
Female sex, *n* (%)	233 (35.2%)	217 (32.8%)	0.051
White Caucasian, *n* (%)	587 (88.8%)	586 (88.7%)	0.005
Comorbidities, *n* (%)			
Hypertension	462 (69.9%)	455 (68.8%)	0.023
Hypercholesterolaemia	352 (53.3%)	330 (49.9%)	0.067
Coronary artery disease	369 (55.8%)	389 (58.9%)	0.061
Diabetes mellitus	225 (34.0%)	207 (31.3%)	0.058
Heart failure	236 (35.7%)	242 (36.6%)	0.019
Chronic obstructive pulmonary disease	104 (15.4%)	105 (15.9%)	0.004
Previous stroke	45 (6.8%)	46 (7.0%)	0.006
Peripheral vascular disease	49 (7.4%)	44 (6.7%)	0.030
Prior gastrointestinal haemorrhage	42 (6.4%)	45 (6.8%)	0.018
Prior intracerebral haemorrhage	12 (1.8%)	16 (2.4%)	0.042
Medications, *n* (%)			
Anticoagulants			
Apixaban	NA	340 (51.4%)	NA
Dabigatran		99 (15.0%)	
Edoxaban		10 (1.5%)	
Rivaroxaban		230 (34.8%)	
Antiplatelets			
Aspirin	163 (24.7%)	165 (25.0%)	0.007
Clopidogrel	45 (6.8%)	42 (6.4%)	0.018
Beta-blockers	206 (31.2%)	202 (30.6%)	0.013
Calcium channel blockers	114 (17.2%)	101 (15.3%)	0.053
Anti-arrhythmic drugs	159 (24.1%)	172 (26.0%)	0.045
ACE-inhibitors	92 (13.9%)	86 (13.0%)	0.027
ARB	56 (8.5%)	58 (8.8%)	0.011
Diuretics	154 (23.3%)	160 (24.2%)	0.021

*ACE* angiotensin-converting enzyme, *ARB* angiotensin receptor blocker, *SD* standard deviation, *SMD* standardised mean difference

### Clinical outcomes before PSM (unadjusted)

The follow-up duration was comparable between both groups. At 2 years, the risk of all-cause mortality was significantly lower in the LAA occlusion group (1.43%) compared to the NOAC group (4.41%) (sTable 1). There were no significant differences in terms of the risk of composite thrombotic or thromboembolic events, or ischaemic stroke or TIA. The risk of venous thromboembolism was reduced with LAA occlusion though these patients were at an increased risk of intracranial haemorrhage.

### Clinical outcomes after PSM

At 2 years, the risk of all-cause mortality was significantly lower among patients who received LAA occlusion compared to NOAC therapy [1.51% vs. 5.60%, RR 0.27 (95% CI 0.14–0.54)], with no statistical difference in terms of the composite thrombotic or thromboembolic events [8.17% vs. 7.72%, RR 1.06 (95% CI 0.73–1.53)], ischaemic stroke or TIA [4.69% vs. 5.45%, RR 0.86 (95% CI 0.54–1.38)], venous thromboembolism [1.66% vs. 1.51%, RR 1.10 (95% CI 0.47–2.57)] and intracranial haemorrhage [1.51% vs. 1.51%, RR 1.00 (95% CI 0.42–2.39)] (Table 3). Kaplan–Meier survival analysis curves with the respective log-rank values are shown in sFigure 1.

**Table 3:** Long-term outcomes with LAA occlusion vs. NOAC therapy after propensity score matching

	LAA occlusion (*n* = 661)		NOAC (*n* = 661)		Risk difference, % (95% CI)	*p* value	Relative risk, % (95% CI)
	*n*	Risk (%)	*n*	Risk (%)			
All-cause mortality	10	1.51	37	5.60	−4.09 (−6.07–(−2.10))	< 0.001	0.27 (0.14–0.54)
Composite thrombotic or thromboembolic events	54	8.17	51	7.72	0.45 (−2.46–3.37)	0.760	1.06 (0.73–1.53)
Ischaemic stroke or TIA	31	4.69	36	5.45	−0.76 (−3.12–1.61)	0.531	0.86 (0.54–1.38)
Venous thromboembolism	11	1.66	10	1.51	0.15 (−1.20–1.50)	0.826	1.10 (0.47–2.57)
Intracranial haemorrhage	10	1.51	10	1.51	0 (−1.32–1.32)	1.000	1.00 (0.42–2.39)

*CI* confidence interval, *LAA* left atrial appendage, *NOAC* non-vitamin K antagonist oral anticoagulant, *TIA* transient ischaemic attack

### Analysis of PSM cohorts with LAA occlusion vs. VKA

In a separate analysis of PSM cohorts with LAA occlusion vs. VKA (sTable 2), we found that LAA occlusion was associated with a significant reduction in all-cause mortality [1.46% vs. 6.85%, RR 0.21 (95% CI 0.11–0.42)], composite thrombotic or thromboembolic events [7.87% vs. 12.97%, RR 0.61 (95% CI 0.44–0.84)], and ischaemic stroke or TIA [4.52% vs. 7.14%, RR 0.63 (95% CI 0.41–0.98)] (Table 4). There were no statistical differences between the groups for venous thromboembolism [1.60% vs. 3.06%, RR 0.52 (95% CI 0.25–1.08)] and intracranial haemorrhage [1.46% vs. 1.75%, RR 0.83 (95% CI 0.36–1.92)].

**Table 4:** Long-term outcomes with LAA occlusion vs. VKA therapy after propensity score matching

	LAA occlusion (*n* = 686)		VKA (*n* = 686)		Risk difference, % (95% CI)	*p* value	Relative risk, % (95% CI)
	*n*	Risk (%)	*n*	Risk (%)			
All-cause mortality	10	1.46	47	6.85	−5.39 (−7.49–(−)3.30)	< 0.001	0.21 (0.11–0.42)
Composite thrombotic or thromboembolic events	54	7.87	89	12.97	−5.10 (−8.32–(−)1.88)	0.002	0.61 (0.44–0.84)
Ischaemic stroke or TIA	31	4.52	49	7.14	−2.62 (−5.10–(-)0.15)	0.038	0.63 (0.41–0.98)
Venous thromboembolism	11	1.60	21	3.06	−1.46 (−3.05–0.14)	0.074	0.53 (0.25–1.08)
Intracranial haemorrhage	10	1.46	12	1.75	−0.29 (−1.62–1.04)	0.667	0.83 (0.36–1.92)

*CI* confidence interval, *LAA* left atrial appendage, *TIA* transient ischaemic attack, *VKA* vitamin K antagonist

## Discussion

In this study, we compared the long-term outcomes of LAA occlusion against NOAC therapy in a large cohort of patients with AF across several health-care organisations, mainly from the USA. The major findings were that patients with AF who received LAA occlusion over NOAC therapy: (1) were younger, more likely to be white Caucasian and male with a greater incidence of comorbidities; (2) were prescribed less cardiovascular medications; (3) had a significantly reduced risk of all-cause mortality at 2-year follow-up; (4) had no statistical difference in the long-term outcomes of the composite of thrombotic or thromboembolic events, ischaemic stroke or TIA, venous thromboembolism, and intracranial haemorrhage. We also demonstrated that LAA occlusion was associated with a reduction in all-cause mortality compared to VKA. Moreover, there was a significant benefit of LAA occlusion over VKA, in terms of the composite thrombotic or thromboembolic events, and ischaemic stroke or TIA which was not observed in the comparison between LAA occlusion and NOAC therapy.

The baseline characteristics of patients who underwent LAA occlusion in this study were similar to that of both the PROTECT AF and PREVAIL RCTs which recruited patients who were eligible for warfarin [10, 11], indicating appropriate patient selection in this real-world cohort. Overall, there was a very low incidence of prior major bleeding in both groups, especially intracranial haemorrhage. In contrast, the incidence of prior major bleeding was much higher (> 70%) in other real-world studies that were limited to the use of LAA occlusion in patients who had contraindications to systemic anticoagulation [12, 13]. This may partly explain the low rates of mortality at 2 years. Despite a greater burden of comorbidities, we observed that patients who received LAA occlusion were prescribed far fewer cardiovascular medications. This may suggest a degree of intolerance to medical therapy, thereby contributing to the indication for LAA occlusion in the first instance. However, the possibility of residual confounders cannot be excluded with this observational study design.

We demonstrated that patients with AF who received LAA occlusion had a significantly reduced risk of all-cause mortality compared to those treated with NOAC. Initial differences in the risk of venous thromboembolism and intracranial haemorrhage between the groups were due to disparities in baseline characteristics, or confounding by indication as those perceived to be at higher risk of intracranial haemorrhage are often referred for LAA occlusion. Furthermore, both groups had a comparable risk of thrombotic and thromboembolic complications, and ischaemic stroke or TIA. The exact mechanisms in which LAA occlusion offers prognostic mortality benefit remains undetermined and could merely be a chance finding. Unfortunately, we were unable to delineate between cardiovascular and non-cardiovascular causes of death.

A study of high-risk patients with AF enrolled in the Amulet Observation Registry reported that patients who were treated with LAA occlusion had a significantly lower risk of the composite outcome of ischaemic stroke, major bleeding or all-cause mortality compared to those who received NOAC therapy over a 2-year follow-up period [14]. In contrast, but similar to our findings, the PRAGUE-17 trial which remains the only randomised controlled trial to directly compare the effects of LAA occlusion vs. NOAC therapy, demonstrated no significant difference in the composite outcome of stroke/TIA and clinically significant bleeding between either treatment [9]. This study was limited to patients with a history of major bleeding, resistant stroke, or moderate- to high-risk profile by CHA_2_DS_2_-VASc and HAS-BLED scores. A network meta-analysis of 14 studies with 246,005 patients also found no significant differences in outcomes between LAA occlusion and NOAC therapy but relied on indirect comparisons to arrive at that conclusion [15]. Further studies are needed to investigate the possible prognostic benefit offered by LAA occlusion found in our study. In this regard, the CATALYST (NCT04226547), Occlusion-AF (NCT03642509) and CLOSURE-AF (NCT03463317) trials may provide useful information but are not due to be completed until the end of this decade.

Apart from all-cause mortality, long-term outcomes in this cohort were comparable to other studies. In PROTECT AF, the observed rate of all-cause mortality was 3.0 (95% CI 1.9–4.5) per 100 patient-years (PYs), ischaemic stroke was 2.2 (95% CI 1.2–3.5) per 100 PYs and haemorrhagic stroke was 0.1 (95% CI 0–0.5) per 100 PYs in the intervention arm compared to 4.8 (95% CI 2.8–7.1) per 100 PYs, 1.6 (95% CI 0.6–3.0) per 100 PYs and 1.6 (95% CI 0.6–3.1) per 100 PYs in the warfarin arm, respectively [10]. During the 2-year follow-up, the EWOLUTION registry found that LAA occlusion was associated with an all-cause mortality of 16.4% (95% CI 13.8–19.3%), stroke rate of 1.3 (95% CI 0.8–1.9) per 100 PYs and major bleeding rate of 2.7 (95% CI 2.0–3.6) per 100 PYs [16]. A real-world observational study of LAA occlusion by Tzikas et al*.* reported 1-year outcomes in terms of all-cause mortality, systemic thromboembolism and major bleeding of 4.3%, 2.3% and 2.1%, respectively [17]. In the ASAP study, the annual risks of all-cause mortality, ischaemic stroke and haemorrhagic stroke were 5.0%, 1.7% and 0.6%, respectively [18].

### Limitations

There are several limitations to this study. Much of the collected data were based on ICD codes from electronic medical records which may vary by patient characteristics and between different health-care organisations [19]. There was also likely a degree of selection bias as we found that patients who received LAA occlusion had a greater incidence of comorbidities. To account for this, we used statistical adjustments and PSM for known confounders. However, we cannot exclude the possibility of residual confounders. The incidence of major bleeding prior to LAA occlusion was low in this cohort and therefore our results may not be applicable to such patients. Furthermore, we did not have data relating to the indication for LAA occlusion, post-LAA occlusion therapy and exact cause of death. As our cohort of patients were comprised of predominantly white Caucasian males, the findings may not be generalisable to the wider population.

## Conclusions

Overall, LAA occlusion might be a suitable alternative to NOAC therapy for stroke prevention in low-risk patients with AF and appears to be associated with good long-term outcomes. However, appropriate patient selection remains an integral aspect of this treatment. Further studies are needed to confirm our findings.

## Supplementary Information

Below is the link to the electronic supplementary material.

Supplementary file1 (DOCX 129 KB)

## Data Availability

Not applicable.
