# Supplementary material for: Outcomes of left atrial appendage occlusion vs. non-vitamin K antagonist oral anticoagulants in atrial fibrillation
Source: Clin Res Cardiol. 2022 Jan 7;111(9):1040–7. doi: 10.1007/s00392-021-01983-z (PMC9424138; doi:10.1007/s00392-021-01983-z)
Supplement: Supplementary file 1 — Supplementary file1 (DOCX 129 KB) [file 392_2021_1983_MOESM1_ESM.docx]

Outcomes of left atrial appendage occlusion *vs.* non-vitamin K antagonist oral anticoagulants in atrial fibrillation

Short title: LAA occlusion *vs.* NOAC therapy in AF

Wern Yew Ding^a^; José Miguel Rivera-Caravaca^a,b,c^; Elnara Fazio-Eynullayeva^d^; Paula Underhill^e^; Dhiraj Gupta^a^; Francisco Marín^b^; Gregory Y. H. Lip^a,f^

^a^Liverpool Centre for Cardiovascular Science, University of Liverpool and Liverpool Heart & Chest Hospital, Liverpool, United Kingdom; ^b^Department of Cardiology, Hospital Clínico Universitario Virgen de la Arrixaca, University of Murcia, Instituto Murciano de Investigación Biosanitaria (IMIB-Arrixaca), CIBERCV, Murcia, Spain; ^c^Liverpool University Hospitals NHS Foundation Trust, Liverpool, United Kingdom; ^d^TriNetX LLC., Cambridge, MA, United States; ^e^TriNetX LLC., London, United Kingdom; ^f^Aalborg Thrombosis Research Unit, Department of Clinical Medicine, Aalborg University, Aalborg, Denmark.

Corresponding author:

Prof Gregory Y H Lip [gregory.lip@liverpool.ac.uk](mailto:gregory.lip@liverpool.ac.uk)

Full mailing address University of Liverpool

William Henry Duncan Building

6 West Derby Street

Liverpool L7 8TX, United Kingdom

Telephone number 0151 794 9020

# Supplement 1: Standardisation of data

TriNetX standardises data, which have been extracted from EMR systems, in two ways:

Standardizing the structure, i.e., putting data into a common structure or data model

TriNetX data providers deliver data in any of a variety of formats: Informatics for Integrating Biology and the Bedside (i2b2), Observational Medical Outcomes Partnership (OMOP), TriNetX ingestion file specification, the North American Association of Central Caner Registries (NAACCR) tumour registry structure, and others. TriNetX have product capabilities that have been tested extensively that map data from each of these structures to the common model within TriNetX.

Standardizing the terminology, i.e., mapping codes, units, etc, to a consistent standard

TriNetX have a team of Informaticists that map data from the data provider’s local codes to master terminology within TriNetX. For example, TriNetX map the provider’s medication codes to RxNorm medication codes. TriNetX map the provider’s local laboratory codes to LOINC laboratory codes.To quality check that mapping, TriNetX review the mapping with the data provider, and TriNetX run data quality tools to identify anomalies and outliers that may indicate mappings that need to be changed. In the event that identify issues are identified within the mapping, the mapping is fixed.

There are occasions when data providers provide data that shows anomalies (e.g., an unusually high lab value, two encounters with the same encounter ID). In other words, these are issues not with our standardization of the data by TriNetX, but with the source data itself. In general, TriNetX hesitate to change source data values because changing the data would impose one interpretation of the data unilaterally. TriNetX prefer to give the data providers flexibility to interpret the data based on their own use case. When TriNetX find any anomalies, the data providers are notified so that they can determine whether they have introduced an issue in their own data processing vs. whether the source data are consistent with what they provided to TriNetX.

# Supplement 2: Codes (ICD-10 and others)

|  | **Codes (ICD-10 and others)** |
| --- | --- |
| **Comorbidities** |  |
| Acute rheumatic fever | I00-I02 |
| Atrial fibrillation | I48 |
| Chronic obstructive pulmonary disease | J44 |
| Coronary artery disease | I25 |
| Diabetes mellitus | E08-E13 |
| Gastrointestinal haemorrhage | K92.2 |
| Heart failure | I50 |
| Hypercholesterolaemia | E78.5 |
| Hypertension | I10 |
| Peripheral vascular disease | I73.9 |
| Previous intracerebral haemorrhage | I61 |
| Previous stroke | I63 |
| Rheumatic heart disease | I05-I09 |
| **Medications** |  |
| ACE-inhibitors | CV800 |
| Anti-arrhythmics | CV300 |
| Apixaban | 1364430 |
| ARB | CV150 |
| Aspirin | 1191 |
| Beta-blockers | CV100 |
| Calcium channel blockers | CV200 |
| Clopidogrel | 32968 |
| Dabigatran | 1037042 |
| Diuretics | CV700 |
| Edoxaban | 1599538 |
| Rivaroxaban | 1114195 |
| **Outcomes** |  |
| Composite thrombotic and thromboembolic events | G45, I63, I67.82, I60, I61, I62, I26, I81, I21, I22, I74 |
| Intracranial haemorrhage | I60, I61, I62 |
| Ischaemic stroke/TIA | G45, I63, I67.82 |
| Venous thromboembolism | I26, I81, I22 |

ACE, angiotensin-converting enzyme; ARB, angiotensin-receptor blocker, ICD; International Classification of Diseases; TIA, transient ischaemic attack.

# Supplement 3: Tables and Figure

**sTable 1**. Long-term outcomes with LAA occlusion *vs.* NOAC *before* propensity score matching

|  | LAA occlusion  (n = 699) | | NOAC  (n = 107,998) | | Risk difference, % (95% CI) | *p* value | Relative risk, % (95% CI) |
| --- | --- | --- | --- | --- | --- | --- | --- |
|  | n | Risk (%) | n | Risk (%) |  |  |  |
| All-cause mortality | 10 | 1.43 | 4,759 | 4.41 | -2.98 (-4.05 - (-)2.25) | <0.001 | 0.31 (0.17 - 0.58) |
| Composite thrombotic or thromboembolic events | 56 | 8.01 | 8,138 | 7.53 | 0.48 (-2.28 - 1.80) | 0.815 | 1.03 (0.80 - 1.33) |
| Ischaemic stroke or TIA | 34 | 4.86 | 4,093 | 3.79 | 1.07 (-2.8 - 0.65) | 0.195 | 1.25 (0.89 - 1.73) |
| Venous thromboembolism | 11 | 1.57 | 3,183 | 2.95 | -1.38 (-2.53 - (-)0.73) | 0.013 | 0.47 (0.25 - 0.84) |
| Intracranial haemorrhage | 10 | 1.43 | 655 | 0.61 | 0.81 (-1.71 - 0.08) | 0.007 | 2.32 (1.23 - 4.35) |

CI, confidence interval; LAA, left atrial appendage; NOAC, non-vitamin K antagonist oral anticoagulant; TIA, transient ischaemic attack.

**sTable 2**. Baseline characteristics of patients with LAA occlusion *vs.* NOAC therapy *after* propensity score matching

|  | LAA occlusion  (n = 661) | NOAC  (n = 661) | SMD |
| --- | --- | --- | --- |
| Age (years), mean (±SD) | 69.9 ± 10.8 | 69.2 ± 12.6 | 0.063 |
| Female sex, n (%) | 233 (35.2%) | 217 (32.8%) | 0.051 |
| White Caucasian, n (%) | 587 (88.8%) | 586 (88.7%) | 0.005 |
| Comorbidities, n (%) |  |  |  |
| Hypertension | 462 (69.9%) | 455 (68.8%) | 0.023 |
| Hypercholesterolaemia | 352 (53.3%) | 330 (49.9%) | 0.067 |
| Coronary artery disease | 369 (55.8%) | 389 (58.9%) | 0.061 |
| Diabetes mellitus | 225 (34.0%) | 207 (31.3%) | 0.058 |
| Heart failure | 236 (35.7%) | 242 (36.6%) | 0.019 |
| Chronic obstructive pulmonary disease | 104 (15.4%) | 105 (15.9%) | 0.004 |
| Previous stroke | 45 (6.8%) | 46 (7.0%) | 0.006 |
| Peripheral vascular disease | 49 (7.4%) | 44 (6.7%) | 0.030 |
| Prior gastrointestinal haemorrhage | 42 (6.4%) | 45 (6.8%) | 0.018 |
| Prior intracerebral haemorrhage | 12 (1.8%) | 16 (2.4%) | 0.042 |
| Medications, n (%) |  |  |  |
| Anticoagulants |  |  |  |
| Apixaban | NA | 340 (51.4%) | NA |
| Dabigatran |  | 99 (15.0%) |  |
| Edoxaban |  | 10 (1.5%) |  |
| Rivaroxaban |  | 230 (34.8%) |  |
| Antiplatelets |  |  |  |
| Aspirin | 163 (24.7%) | 165 (25.0%) | 0.007 |
| Clopidogrel | 45 (6.8%) | 42 (6.4%) | 0.018 |
| Beta-blockers | 206 (31.2%) | 202 (30.6%) | 0.013 |
| Calcium channel blockers | 114 (17.2%) | 101 (15.3%) | 0.053 |
| Anti-arrhythmic drugs | 159 (24.1%) | 172 (26.0%) | 0.045 |
| ACE-inhibitors | 92 (13.9%) | 86 (13.0%) | 0.027 |
| ARB | 56 (8.5%) | 58 (8.8%) | 0.011 |
| Diuretics | 154 (23.3%) | 160 (24.2%) | 0.021 |

ACE, angiotensin-converting enzyme; ARB, angiotensin receptor blocker; SD, standard deviation; SMD, standardised mean difference.

**sTable 3**. Baseline characteristics of patients with LAA occlusion *vs.* VKA therapy *after* propensity score matching

|  | LAA occlusion  (n = 686) | VKA  (n = 686) | SMD |
| --- | --- | --- | --- |
| Age (years), mean (±SD) | 70.0 ± 10.7 | 69.5 ± 13.5 | 0.042 |
| Female sex, n (%) | 234 (34.1%) | 236 (34.4%) | 0.006 |
| White Caucasian, n (%) | 613 (89.4%) | 607 (88.5%) | 0.028 |
| Comorbidities, n (%) |  |  |  |
| Hypertension | 485 (70.7%) | 490 (71.4%) | 0.016 |
| Hypercholesterolaemia | 371 (54.1%) | 365 (53.2%) | 0.018 |
| Coronary artery disease | 64 (9.3%) | 66 (9.6%) | 0.010 |
| Diabetes mellitus | 243 (35.4%) | 232 (33.8%) | 0.034 |
| Heart failure | 253 (36.9%) | 247 (36.0%) | 0.018 |
| Chronic obstructive pulmonary disease | 108 (15.7%) | 119 (17.4%) | 0.043 |
| Previous stroke | 50 (7.3%) | 62 (9.0%) | 0.064 |
| Peripheral vascular disease | 53 (7.7%) | 59 (8.6%) | 0.032 |
| Prior gastrointestinal haemorrhage | 44 (6.4%) | 49 (7.1%) | 0.029 |
| Prior intracerebral haemorrhage | 14 (2.0%) | 21 (3.1%) | 0.065 |
| Medications, n (%) |  |  |  |
| Antiplatelets |  |  |  |
| Aspirin | 182 (26.5%) | 206 (30.0%) | 0.078 |
| Clopidogrel | 50 (7.3%) | 62 (9.0%) | 0.064 |
| Beta-blockers | 209 (30.5%) | 210 (30.6%) | 0.003 |
| Calcium channel blockers | 108 (15.7%) | 109 (15.9%) | 0.004 |
| Anti-arrhythmic drugs | 156 (22.7%) | 166 (24.2%) | 0.034 |
| ACE-inhibitors | 94 (13.7%) | 88 (12.8%) | 0.026 |
| ARB | 59 (8.6%) | 55 (8.0%) | 0.021 |
| Diuretics | 154 (22.5%) | 175 (25.5%) | 0.072 |

ACE, angiotensin-converting enzyme; ARB, angiotensin receptor blocker; SD, standard deviation; SMD, standardised mean difference.


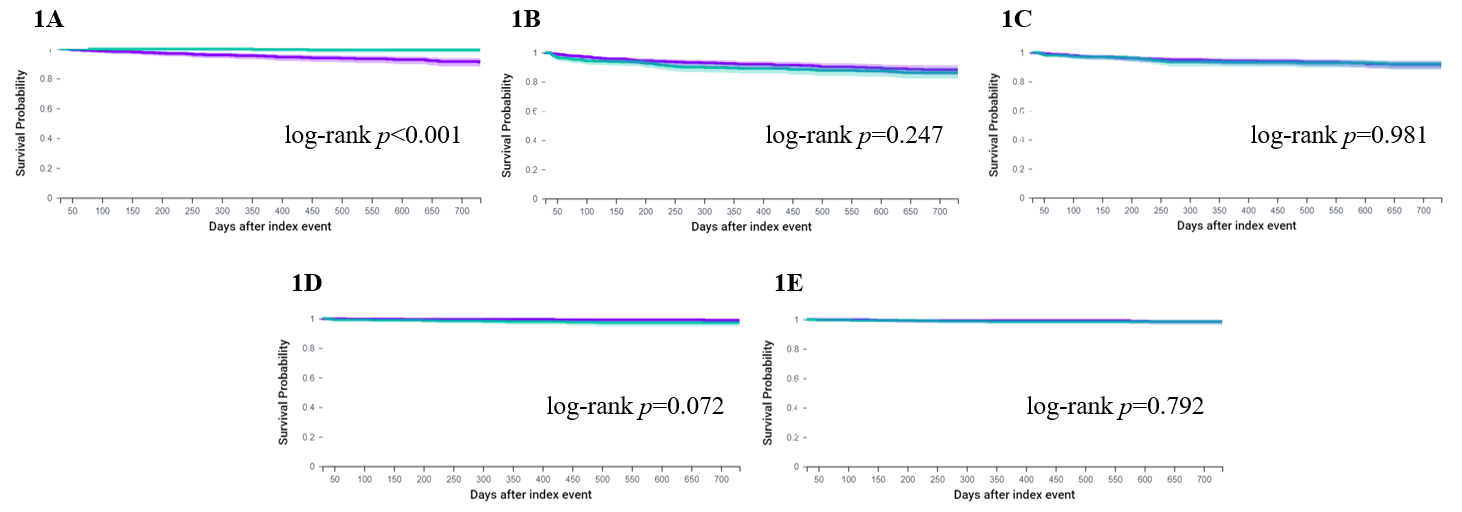


**sFigure 1**: Kaplan-Meier curves for all-cause mortality (1A), composite of thrombotic and thromboembolic events (1B), ischaemic stroke or TIA (1C), venous thromboembolism (1D), and intracranial haemorrhage (1E). TIA, transient ischaemic attack.
